# RUMINATION, DEPRESSIVE SYMPTOMS, AND SLEEP QUALITY: SOCIAL SUPPORT AS A BUFFER

**DOI:** 10.1093/geroni/igz038.1940

**Published:** 2019-11-08

**Authors:** Christina M Marini, Stephanie J Wilson, Lynn Martire

**Affiliations:** 1 The Center for Healthy Aging at The Pennsylvania State University, University Park, Pennsylvania, United States; 2 The Ohio State University College of Medicine, Columbus, Ohio, United States; 3 The Pennsylvania State University, University Park, Pennsylvania, United States

## Abstract

Rumination, the act of dwelling on negative, unwanted thoughts, can stoke depression and disrupt sleep, both of which may threaten older adults’ well-being. In line with a support buffering hypothesis, a previous study of younger and middle-aged adults found that social support mitigated the positive association between rumination and negative mood. To extend this research, we distinguished between spousal and family/friend support as moderators of rumination’s links both to depressive symptoms and sleep quality among older adults. Data came from a sample of 128 adults who were, on average, 77 years old at study onset. Rumination was measured via the Rumination-Reflection Questionnaire. Perceived support was measured by items utilized in multiple nationally representative studies of older adults. Depressive symptoms were measured via the NIH PROMIS measure, and sleep quality was measured via items from the Pittsburgh Sleep Quality Index. Results indicated that support from family/friends (but not spouses) buffered the positive association between rumination and depressive symptoms, even after controlling for depressive symptoms six months prior. Conversely, when sleep quality served as the outcome, support from spouses (but not family/friends) buffered the negative association between rumination and sleep quality, even after controlling for sleep quality six months prior. Findings highlight the potential for specific sources of social support to buffer different consequences of rumination on older adults’ health and well-being.

